# (4-Butyl-1-methyl-1,2,4-triazol-5-yl­idene)[(1,2,5,6-η)-cyclo­octa-1,5-diene](tri­phenyl­phosphane)iridium(I) tetra­fluorido­borate

**DOI:** 10.1107/S2414314621011421

**Published:** 2021-11-04

**Authors:** Kassandra T. Castaldi, Andrei V. Astashkin, Daniel R. Albert, Edward Rajaseelan

**Affiliations:** a Department of Chemistry, Millersville University, Millersville PA, 17551, USA; b Department of Chemistry and Biochemistry, The University of Arizona, Tuscon, AZ, 85716, USA; Vienna University of Technology, Austria

**Keywords:** crystal structure, iridium, N-heterocyclic carbenes, cationic complexes

## Abstract

A new triazole-based N-heterocyclic cationic carbene iridium(I) complex with a tetra­fluorido­borate counter-anion, [Ir(C_8_H_12_)(C_7_H_13_N_3_)(C_18_H_15_P)]BF_4_, has been synthesized and structurally characterized. The Ir^I^ atom of the cationic complex has an expected square-planar coordination environment with unexceptional bond lengths. There are several close F⋯H contacts between the cations and the anions in the range 2.36–2.58 Å, stabilizing the orientation of the out-sphere [BF_4_^−^] counter-anion. In the crystal, C—H⋯π(ring) inter­actions are observed that orient the phenyl rings of the tri­phenyl­phosphane ligands.

## Abstract 2

In the title compound, the iridium(I) atom has a distorted square-planar coordination environment.

## Graphical abstract

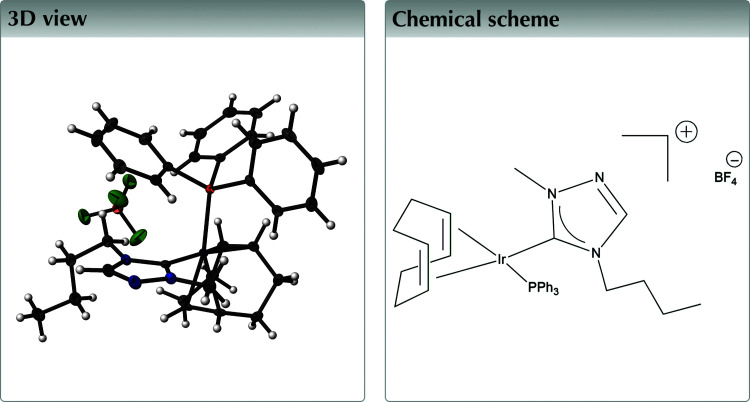

## Structure description

N-heterocyclic carbenes (NHC) have become important alternatives to phosphanes as ancillary ligands in transition-metal chemistry, synthesis, and in homogeneous catalysis (Cazin, 2013 ▸; Díez-Gonzáles *et al.*, 2009 ▸; Rovis & Nolan, 2013 ▸; Ruff *et al.*, 2016 ▸; Zuo *et al.*, 2014 ▸). Their catalytic activities in the transfer hydrogenation of ketones and imines have also been studied and reported (Albrecht *et al.*, 2002 ▸; Gnanamgari *et al.*, 2007 ▸). NHC ligands can be tuned sterically and electronically by having different substituents on the nitro­gen atoms (Gusev, 2009 ▸). Although many imidazole- and triazole-based NHC rhodium and iridium complexes have been prepared and structurally characterized (Herrmann *et al.*, 2006 ▸; Wang & Lin, 1998 ▸; Chianese *et al.*, 2004 ▸), new imidazole and triazole-based NHC complexes of rhodium and iridium are still being synthesized to study the effect of different substituents on NHC ligands and other ligands coordinating to the metal in transfer hydrogenation reactions (Nichol *et al.*, 2009 ▸, 2010 ▸, 2011 ▸, 2012 ▸; Idrees *et al.*, 2017*a* ▸,*b* ▸; Rood *et al.*, 2021 ▸; Rushlow *et al.*, 2021 ▸; Newman *et al.*, 2021 ▸).

**Figure 1:**
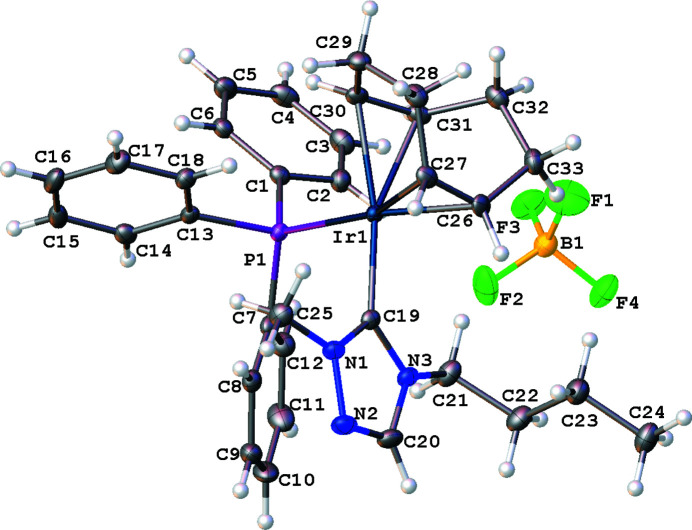
The mol­ecular entities in the crystal structure of the title compound (**4**). Displacement ellipsoids are drawn at the 50% probability level.

**Figure 2:**
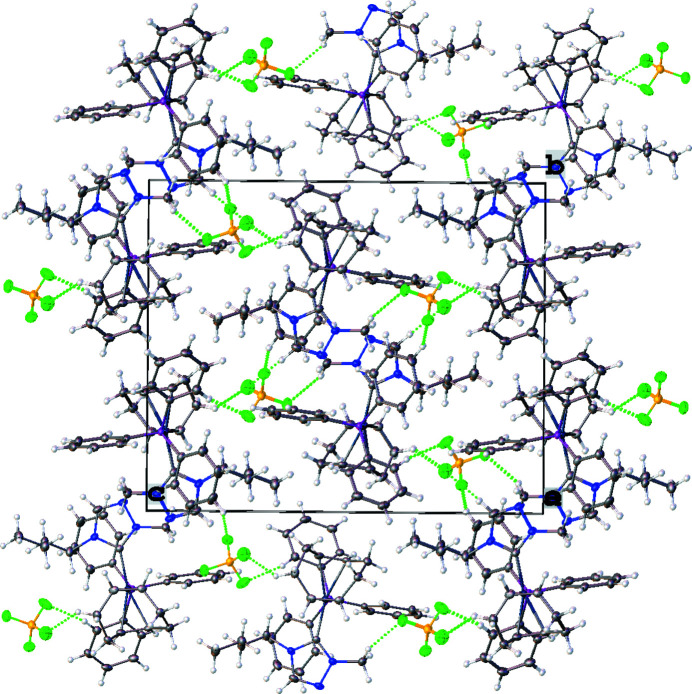
Crystal packing unit-cell diagram of the title compound (**4**) shown along the *a* axis. Hydrogen-bonding inter­actions between F and H atoms are shown as dotted green lines. Add axis labels

**Figure 3:**
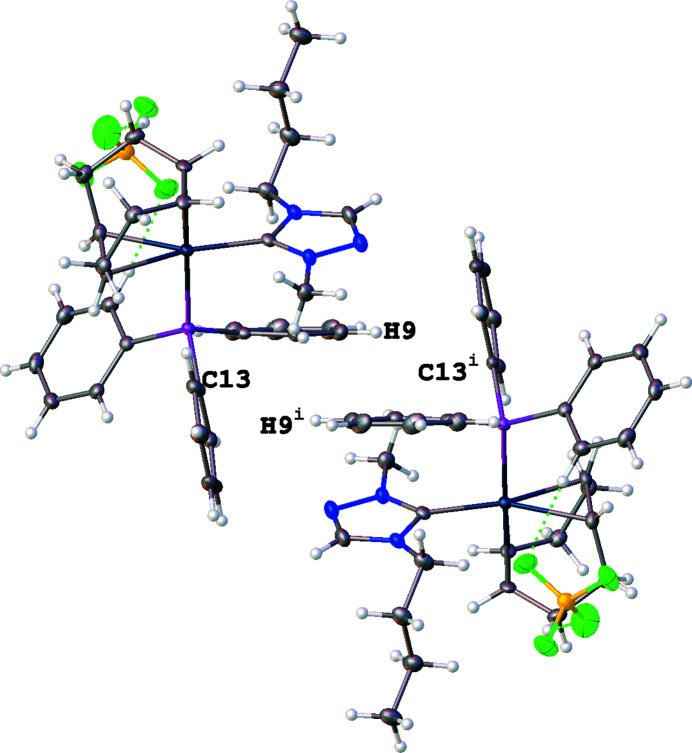
View of the title compound (**4**) showing T-shaped distorted perpendicular inter­actions arising from C—H⋯π(ring) inter­actions between a hydrogen atom on a phenyl ring (H9) and a phenyl ring (C13–C18) of tri­phenyl­phosphane. [Symmetry code: (i) −*x* + 2, −*y* + 1, −*z* + 1].

The mol­ecular structure of the title complex, [Ir(C_8_H_12_)(C_18_H_15_P)(C_7_H_13_N_3_)][BF_4_] (**4**), comprises an Ir^I^ cationic complex and a tetra­fluorido­borate counter-anion, illustrated in Fig. 1 ▸. The coordination sphere around the Ir^I^ atom, formed by the bidentate cyclo­octa-1,5-diene (COD), the carbene C atom of the NHC, and the P atom of the tri­phenyl­phosphane ligand, exhibits a distorted square-planar geometry. The carbene atom, C19, deviates from the expected *sp* ^2^ hybridization in that the N1—C19—N3 bond angle in the triazole-based carbene is 103.41 (18)°. Other selected bond lengths and angles in the structure are: Ir1—C19_(NHC)_ = 2.043 (2) Å, Ir1—P1 = 2.3330 (6) Å, and C19—Ir1—P1 = 91.94 (6)°. Fig. 2 ▸ shows the molecular packing diagram of the complex (**4**). There are several close F⋯H contacts (likely, non-standard hydrogen bonds between the cation and anion), stabilizing the orientation of the [BF_4_^−^] group as reported in Table 1 ▸ and shown as dotted green lines in Fig. 2 ▸. An intra­molecular C—H⋯π(ring) inter­action is observed between a hydrogen atom on the butyl wingtip of the NHC (H21*B*) and a phenyl phosphane ring (C7–C12) with an H⋯centroid distance of 2.84 Å and a C—H⋯centroid angle of 139°. Inter­molecular C—H⋯π(ring) inter­actions are observed between phenyl phosphane rings on adjacent moieties with a hydrogen atom of a phenyl ring (H9) inter­acting with a phenyl phosphane ring (C13–C18). The inter­molecular C—H⋯π(ring) inter­action has an H⋯centroid distance of 2.77 Å and a C—H⋯centroid angle of 159°. The inter­action results in nearly perpendicular T-shaped orientations of the phenyl rings (C7–C12 and C13–C18), as seen in Fig. 3 ▸, with a dihedral angle of 80.43 (11)° between the ring planes.

## Synthesis and crystallization

**Figure 4:**
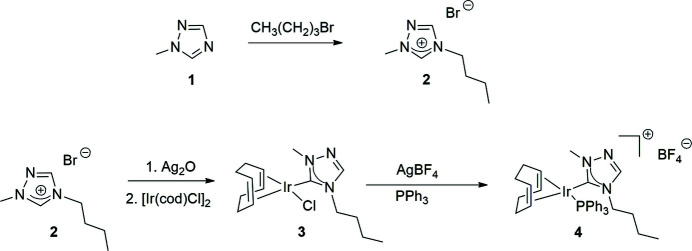
Reaction scheme for the synthesis of the N-heterocyclic carbene (**2**) and subsequent formation of the title compound (**4**).

**1-Methyl-1,2,4-triazole** (**1**) was purchased from Matrix Scientific. All other compounds used in the syntheses, shown in Fig. 4 ▸, were obtained from Sigma–Aldrich and Strem and used as received; all syntheses were performed under a nitro­gen atmosphere. NMR spectra were recorded at room temperature in CDCl_3_ on a 400 MHz (operating at 162 MHz for ^31^P) Varian spectrometer and referenced to the residual solvent peak (δ in ppm).

**4-Butyl-1-methyl-1,2,4-triazolium bromide (2):** 1-Methyl-1,2,4-triazole (**1**) (1.231 g, 14.82 mmol) and 1-bromo­butane (3.393 g, 24.76 mmol) were added to toluene (10 mL), and the mixture was refluxed in the dark for 24 h. After the mixture was cooled, the off-white solid was filtered, washed with ether, and dried under vacuum. Yield: 2.228 g (68%). ^1^H NMR: δ 11.42 (*s*, 1 H, N—C_5_H—N), 9.01 (*s*, 1 H, N—C_3_H—N), 4.56 (*t*, 2 H, N—CH­_2_ of *n*-Bu), 4.25 (*s*, 3 H, N—CH_3_), 1.95 (*m*, 2 H, CH_2_ of *n*-Bu), 1.40 (*m*, 2 H, CH_2_ of *n*-Bu), 0.96 (*t*, 3 H, CH_3_ of *n*-Bu). ^13^C NMR: δ 143.77 (N—C_5_—N), 143.35 (N—C_3_—N), 48.60 (N—CH_3_), 39.55 (N-CH­_2_ of *n*-Bu), 31.90 (CH­_2_ of *n*-Bu), 19.43 (CH­_2_ of *n*-Bu), 13.39 (CH­_3_ of *n*-Bu).

**[(1,2,5,6-η)-Cyclo­octa-1,5-diene](4-butyl-1-methyl-1,2,4-tri­azol-5-yl­idene)chloro­iridium (3):** Triazolium bromide (**2**) (0.066 g, 0.300 mmol) and Ag_2_O (0.035 g, 0.151 mmol) were stirred at room temperature in the dark for 1 h in CH_2_Cl_2_ (10 mL). The mixture was then filtered through Celite into [Ir(cod)Cl]_2_ (0.100 g, 0.149 mmol), and stirred again in the dark for 1.5 h. The resulting solution was filtered through Celite and the solvent was removed at reduced pressure. The yellow solid product (**3**) was dried under vacuum. Yield: 0.134g (94%). ^1^H NMR: δ 7.85 (*s*, 1 H, N—C_3_H—N), 4.78 (*t*, 2 H, N—CH_2_ of *n*-Bu), 4.46 (*m*, 2 H, CH of COD), 4.35 (*m*, 2H of COD), 4.14 (*s*, 3 H, N—CH_3_), 3.01, 2.91 (*m*, 4 H, CH_2_ of COD), 2.25,2.09 (*m*, 4 H, CH_2_ of COD), 1.80 (*m*, 2 H, CH_2_ of *n*-Bu), 1.43 (*m*, 2 H, CH_2_ of *n*-Bu), 1.02 (*t*, 3 H, CH_2_ of *n*-Bu). ^13^C NMR: δ 178.56 (Ir—C), 143.73 (N—C_3_H—N), 86.06,85.48, 52.50, 52.10 (CH of COD), 48.80 (N—CH_3_), 48.59 (N- CH­_2_ of *n*-Bu), 33.77,33.20,32.65,32.50 (CH_2_ of COD), 31.35 (CH­_2_ of *n*-Bu), 19.95 (CH­_2_ of *n*-Bu), 13.76 (CH­_3_ of *n*-Bu).

**[(1,2,5,6-η)-Cyclo­octa-1,5-diene](4-butyl-1-methyl-1,2,4-tri­azol-5-yl­idene)(tri­phenyl­phosphane)iridium(I) tetra­fluorido­borate (4):** Tri­phenyl­phosphane (0.074 g, 0.282 mmol) and AgBF_4_ (0.055 g, 0.282 mmol) were added to (**3**) (0.134 g, 0.282 mmol) in CH_2_Cl_2_ (10 mL). The solution was stirred in the dark for 1.5 h. The resulting mixture was filtered through Celite and the solvent was removed at reduced pressure. The bright-orange solid product (**4**) was dried under vacuum. Yield: 0.220 g (99%). ^1^H NMR: δ 8.14 (*s*, 1 H, N-C_3_H-N), 7.26–7.45 (*m*, 15 H, H_ar_), 4.84 (*s*, 3H, N—CH_3_), 4.76 (*t*, 2 H, N—CH_2_ of CH­_2_ of *n*-Bu), 4.52 (*m*, 2 H, CH of COD), 4.36 (*m*, 2H, CH of COD), 3.95 (*m*, 2 H, CH_2_ of COD), 3.84 (*m*, 2 H, CH_2_ of COD), 2.43 (*m*, 2 H, CH_2_ of COD), 2.17 (*m*, 2 H, CH_2_ of COD), 1.55 (*m*, CH_2_ of *n*-Bu), 1.32 (*m*, 2 H, CH_2_ of *n*-Bu), 0.91 (*t*, 3 H, CH_3_ of *n*-Bu). ^13^C NMR: δ 178.27 (Ir—C), 143.44 (N—C_3_H—N), 133.57–129.04 (C arom), 87.66, 87.26, 86.06, 85.18 (CH of COD), 48.48 (N—CH_3_), 39.44 (N—CH­_2_ of *n*-Bu), 33.35, 31.78, 31.18, 30.60 (CH_2_ of COD), 26.23 (CH_2_ of *n*-Bu), 19.98 (CH_2_ of *n*-Bu), 13.65 (CH_3_ of *n*-Bu).^31^P NMR: δ 17.40.

The title compound (**4**) was crystallized by slow diffusion of pentane into a CH_2_Cl­_2_ solution.

## Refinement

Crystal data, data collection and structure refinement details are summarized in Table 2 ▸.

## Supplementary Material

Crystal structure: contains datablock(s) I. DOI: 10.1107/S2414314621011421/wm4155sup1.cif

Structure factors: contains datablock(s) I. DOI: 10.1107/S2414314621011421/wm4155Isup2.hkl

CCDC reference: 2118414

Additional supporting information:  crystallographic information; 3D view; checkCIF report

## Figures and Tables

**Table 1:** Hydrogen-bond geometry (Å, °)

*D*—H⋯*A*	*D*—H	H⋯*A*	*D*⋯*A*	*D*—H⋯*A*
C2—H2⋯F2	0.95	2.36	3.285 (3)	165
C20—H20⋯F1^i^	0.95	2.47	3.329 (3)	150
C25—H25*B*⋯F4^ii^	0.98	2.37	3.245 (3)	149

Symmetry codes: (i) 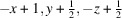 ; (ii) 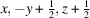 .

**Table 2:** Experimental details

Crystal data	
Chemical formula	[Ir(C_8_H_12_)(C_7_H_13_N_3_)(C_18_H_15_P)]BF_4_
*M*_r_	788.66
Crystal system, space group	Monoclinic, *P*2_1_/*c*
Temperature (K)	100
*a*, *b*, *c* (Å)	9.8966 (9), 16.3247 (17), 20.0560 (19)
β (°)	102.285 (4)
*V* (Å^3^)	3166.0 (5)
*Z*	4
Radiation type	Mo *K*α
μ (mm^−1^)	4.32
Crystal size (mm)	0.31 × 0.21 × 0.19

Data collection	
Diffractometer	Bruker APEXII CCD
Absorption correction	Multi-scan (*SADABS*; Bruker, 2015 ▸)
*T*_min_, *T*_max_	0.668, 0.745
No. of measured, independent and observed [*I* > 2σ(*I*)] reflections	34219, 6457, 5986
*R*_int_	0.028
(sin θ/λ)_max_ (Å^−1^)	0.625

Refinement	
*R*[*F* ^2^ > 2σ(*F* ^2^)], *wR*(*F* ^2^), *S*	0.017, 0.039, 1.02
No. of reflections	6457
No. of parameters	390
H-atom treatment	H-atom parameters constrained
Δρ_max_, Δρ_min_ (e Å^−3^)	0.88, −0.46

Computer programs: *APEX2* and *SAINT* (Bruker, 2015 ▸), *SHELXT* (Sheldrick, 2015*a* ▸), *SHELXL* (Sheldrick, 2015*b* ▸), *OLEX2* (Dolomanov *et al.*, 2009 ▸), and *publCIF* (Westrip, 2010 ▸).

## full crystallographic data

### Crystal data

**Table:**

[Ir(C_8_H_12_)(C_7_H_13_N_3_)(C_18_H_15_P)]BF_4_	*F*(000) = 1568
*M**_r_* = 788.66	*D*_x_ = 1.655 Mg m^−^^3^
Monoclinic, *P*2_1_/*c*	Mo *K*α radiation, λ = 0.71073 Å
*a* = 9.8966 (9) Å	Cell parameters from 9226 reflections
*b* = 16.3247 (17) Å	θ = 2.4–26.4°
*c* = 20.0560 (19) Å	µ = 4.32 mm^−^^1^
β = 102.285 (4)°	*T* = 100 K
*V* = 3166.0 (5) Å^3^	Irregular, clear light pink
*Z* = 4	0.31 × 0.21 × 0.19 mm

### Data collection

**Table:**

Bruker APEXII CCD diffractometer	5986 reflections with *I* > 2σ(*I*)
φ and ω scans	*R*_int_ = 0.028
Absorption correction: multi-scan (SADABS; Bruker, 2015)	θ_max_ = 26.4°, θ_min_ = 1.6°
*T*_min_ = 0.668, *T*_max_ = 0.745	*h* = −9→12
34219 measured reflections	*k* = −20→20
6457 independent reflections	*l* = −25→25

### Refinement

**Table:**

Refinement on *F*^2^	0 restraints
Least-squares matrix: full	Hydrogen site location: inferred from neighbouring sites
*R*[*F*^2^ > 2σ(*F*^2^)] = 0.017	H-atom parameters constrained
*wR*(*F*^2^) = 0.039	*w* = 1/[σ^2^(*F*_o_^2^) + (0.0154*P*)^2^ + 3.1892*P*] where *P* = (*F*_o_^2^ + 2*F*_c_^2^)/3
*S* = 1.02	(Δ/σ)_max_ = 0.002
6457 reflections	Δρ_max_ = 0.88 e Å^−^^3^
390 parameters	Δρ_min_ = −0.46 e Å^−^^3^

### Special details

**Table:**

Geometry. All esds (except the esd in the dihedral angle between two l.s. planes) are estimated using the full covariance matrix. The cell esds are taken into account individually in the estimation of esds in distances, angles and torsion angles; correlations between esds in cell parameters are only used when they are defined by crystal symmetry. An approximate (isotropic) treatment of cell esds is used for estimating esds involving l.s. planes.

### Fractional atomic coordinates and isotropic or equivalent isotropic displacement parameters (Å^2^)

**Table:**

	*x*	*y*	*z*	*U*_iso_*/*U*_eq_	
Ir1	0.65716 (2)	0.26151 (2)	0.45405 (2)	0.01160 (3)	
P1	0.89650 (6)	0.26064 (3)	0.46457 (3)	0.01218 (11)	
F3	0.71162 (17)	0.08389 (9)	0.20651 (8)	0.0358 (4)	
F4	0.55953 (16)	0.17666 (10)	0.14868 (7)	0.0353 (4)	
F1	0.5266 (2)	0.12606 (12)	0.24796 (9)	0.0503 (5)	
F2	0.70664 (17)	0.21312 (10)	0.24647 (9)	0.0433 (4)	
N1	0.65074 (19)	0.44904 (11)	0.47394 (9)	0.0162 (4)	
N3	0.63018 (19)	0.41838 (11)	0.36915 (9)	0.0161 (4)	
N2	0.6419 (2)	0.52330 (12)	0.44004 (10)	0.0211 (4)	
C19	0.6459 (2)	0.38401 (13)	0.43264 (11)	0.0133 (4)	
C14	1.1314 (2)	0.29788 (13)	0.56684 (11)	0.0173 (5)	
H14	1.179953	0.300731	0.530836	0.021*	
C1	0.9725 (2)	0.16434 (13)	0.44288 (11)	0.0142 (4)	
C7	0.9666 (2)	0.33788 (14)	0.41425 (11)	0.0153 (4)	
C30	0.6753 (2)	0.14351 (13)	0.50736 (11)	0.0164 (4)	
H30	0.772530	0.124875	0.525058	0.020*	
C13	0.9890 (2)	0.28236 (13)	0.55206 (10)	0.0140 (4)	
C27	0.4612 (2)	0.26324 (14)	0.49000 (12)	0.0165 (4)	
H27	0.439383	0.317551	0.508224	0.020*	
C18	0.9175 (2)	0.28029 (14)	0.60557 (11)	0.0169 (4)	
H18	0.820762	0.270299	0.596020	0.020*	
C6	1.0729 (2)	0.12075 (13)	0.48883 (11)	0.0166 (4)	
H6	1.102300	0.139853	0.534351	0.020*	
C2	0.9277 (2)	0.13410 (14)	0.37611 (11)	0.0189 (5)	
H2	0.856611	0.161719	0.344911	0.023*	
C29	0.5865 (2)	0.14483 (14)	0.56032 (11)	0.0191 (5)	
H29A	0.639649	0.170571	0.602525	0.023*	
H29B	0.565779	0.087741	0.571466	0.023*	
C31	0.6252 (2)	0.12923 (13)	0.43760 (11)	0.0173 (5)	
H31	0.694465	0.102954	0.414647	0.021*	
C33	0.3877 (2)	0.18133 (15)	0.37746 (12)	0.0208 (5)	
H33A	0.396562	0.191149	0.329864	0.025*	
H33B	0.289372	0.169226	0.376674	0.025*	
C26	0.4292 (2)	0.25843 (14)	0.41893 (12)	0.0175 (5)	
H26	0.388874	0.309840	0.395824	0.021*	
C10	1.0450 (3)	0.46222 (16)	0.33383 (12)	0.0271 (6)	
H10	1.070139	0.504338	0.306101	0.033*	
C8	0.9513 (2)	0.42037 (14)	0.42979 (11)	0.0174 (5)	
H8	0.914969	0.434381	0.468486	0.021*	
C28	0.4490 (2)	0.19174 (14)	0.53677 (11)	0.0190 (5)	
H28A	0.377662	0.153371	0.512567	0.023*	
H28B	0.417828	0.212455	0.577402	0.023*	
C20	0.6288 (2)	0.50151 (14)	0.37636 (12)	0.0209 (5)	
H20	0.619314	0.539019	0.339443	0.025*	
C21	0.6380 (2)	0.37261 (15)	0.30676 (11)	0.0211 (5)	
H21A	0.619985	0.313956	0.313919	0.025*	
H21B	0.733011	0.377231	0.298694	0.025*	
C12	1.0261 (2)	0.31863 (15)	0.35861 (12)	0.0215 (5)	
H12	1.040077	0.262983	0.347945	0.026*	
C11	1.0647 (3)	0.38084 (17)	0.31903 (12)	0.0275 (6)	
H11	1.105034	0.367323	0.281487	0.033*	
C9	0.9881 (2)	0.48205 (15)	0.38964 (12)	0.0220 (5)	
H9	0.974646	0.537806	0.400139	0.026*	
C4	1.0889 (2)	0.02184 (14)	0.40176 (13)	0.0224 (5)	
H4	1.130025	−0.025843	0.387538	0.027*	
C3	0.9873 (2)	0.06395 (14)	0.35580 (12)	0.0214 (5)	
H3	0.958607	0.044591	0.310313	0.026*	
C25	0.6757 (2)	0.44862 (14)	0.54844 (11)	0.0201 (5)	
H25A	0.770805	0.430993	0.567206	0.030*	
H25B	0.611083	0.410699	0.563228	0.030*	
H25C	0.661787	0.503904	0.564823	0.030*	
C5	1.1299 (2)	0.04971 (14)	0.46846 (12)	0.0208 (5)	
H5	1.197231	0.020093	0.500216	0.025*	
C22	0.5356 (2)	0.40274 (16)	0.24331 (11)	0.0227 (5)	
H22A	0.550364	0.462150	0.237934	0.027*	
H22B	0.556200	0.374777	0.202807	0.027*	
C24	0.2888 (3)	0.41824 (17)	0.17845 (12)	0.0273 (6)	
H24A	0.312914	0.390240	0.139350	0.041*	
H24B	0.299661	0.477499	0.173758	0.041*	
H24C	0.192680	0.405874	0.180060	0.041*	
C15	1.2020 (2)	0.30915 (14)	0.63392 (12)	0.0213 (5)	
H15	1.299068	0.318279	0.643787	0.026*	
C23	0.3847 (2)	0.38868 (16)	0.24451 (12)	0.0246 (5)	
H23A	0.361964	0.418257	0.283794	0.029*	
H23B	0.369070	0.329525	0.250673	0.029*	
C16	1.1305 (3)	0.30704 (15)	0.68664 (12)	0.0242 (5)	
H16	1.178888	0.315308	0.732387	0.029*	
C32	0.4761 (2)	0.10623 (14)	0.40586 (12)	0.0200 (5)	
H32A	0.434297	0.079092	0.440757	0.024*	
H32B	0.475337	0.066578	0.368436	0.024*	
B1	0.6273 (3)	0.15018 (17)	0.21277 (13)	0.0199 (5)	
C17	0.9887 (3)	0.29291 (15)	0.67276 (11)	0.0224 (5)	
H17	0.940288	0.291852	0.708901	0.027*	

### Atomic displacement parameters (Å^2^)

**Table:**

	*U*^11^	*U*^22^	*U*^33^	*U*^12^	*U*^13^	*U*^23^
Ir1	0.01031 (5)	0.01204 (5)	0.01236 (4)	0.00107 (3)	0.00225 (3)	0.00077 (3)
P1	0.0116 (3)	0.0132 (3)	0.0115 (2)	0.0014 (2)	0.0020 (2)	0.00072 (19)
F3	0.0445 (10)	0.0277 (8)	0.0356 (9)	0.0155 (7)	0.0094 (7)	0.0015 (7)
F4	0.0410 (9)	0.0387 (9)	0.0223 (8)	0.0116 (7)	−0.0024 (6)	0.0008 (6)
F1	0.0625 (12)	0.0533 (12)	0.0466 (11)	−0.0028 (9)	0.0373 (9)	−0.0002 (8)
F2	0.0370 (9)	0.0358 (9)	0.0489 (10)	0.0014 (8)	−0.0092 (8)	−0.0191 (8)
N1	0.0179 (10)	0.0126 (9)	0.0182 (9)	0.0020 (7)	0.0040 (7)	0.0012 (7)
N3	0.0155 (9)	0.0157 (10)	0.0168 (9)	0.0030 (7)	0.0025 (7)	0.0034 (7)
N2	0.0226 (11)	0.0141 (10)	0.0263 (11)	0.0025 (8)	0.0046 (8)	0.0044 (8)
C19	0.0094 (10)	0.0155 (11)	0.0155 (10)	0.0022 (8)	0.0038 (8)	0.0015 (8)
C14	0.0164 (11)	0.0128 (11)	0.0228 (11)	0.0017 (9)	0.0044 (9)	−0.0002 (9)
C1	0.0118 (10)	0.0141 (11)	0.0176 (11)	−0.0007 (8)	0.0050 (8)	−0.0002 (8)
C7	0.0096 (10)	0.0203 (12)	0.0157 (10)	−0.0006 (9)	0.0017 (8)	0.0036 (8)
C30	0.0156 (11)	0.0113 (11)	0.0223 (11)	0.0026 (9)	0.0042 (9)	0.0024 (8)
C13	0.0154 (11)	0.0108 (10)	0.0145 (10)	0.0013 (8)	0.0005 (8)	0.0008 (8)
C27	0.0100 (10)	0.0167 (11)	0.0237 (11)	0.0015 (9)	0.0053 (9)	−0.0009 (9)
C18	0.0158 (11)	0.0165 (11)	0.0180 (11)	0.0022 (9)	0.0023 (9)	0.0014 (8)
C6	0.0151 (11)	0.0153 (11)	0.0190 (11)	−0.0028 (9)	0.0032 (9)	−0.0004 (8)
C2	0.0159 (11)	0.0210 (12)	0.0191 (11)	−0.0009 (9)	0.0023 (9)	0.0002 (9)
C29	0.0213 (12)	0.0177 (12)	0.0190 (11)	−0.0022 (9)	0.0060 (9)	0.0031 (9)
C31	0.0177 (12)	0.0125 (11)	0.0232 (12)	0.0009 (9)	0.0075 (9)	−0.0003 (8)
C33	0.0164 (12)	0.0255 (13)	0.0192 (11)	−0.0038 (10)	0.0009 (9)	−0.0027 (9)
C26	0.0076 (10)	0.0182 (11)	0.0251 (12)	0.0016 (8)	0.0000 (9)	0.0020 (9)
C10	0.0295 (14)	0.0295 (14)	0.0220 (12)	−0.0114 (11)	0.0045 (10)	0.0085 (10)
C8	0.0130 (11)	0.0202 (12)	0.0182 (11)	0.0002 (9)	0.0015 (8)	0.0011 (9)
C28	0.0172 (12)	0.0213 (12)	0.0201 (11)	−0.0028 (9)	0.0078 (9)	−0.0019 (9)
C20	0.0211 (12)	0.0162 (12)	0.0254 (12)	0.0031 (9)	0.0051 (10)	0.0070 (9)
C21	0.0222 (12)	0.0264 (13)	0.0151 (11)	0.0065 (10)	0.0046 (9)	0.0004 (9)
C12	0.0212 (12)	0.0238 (12)	0.0207 (12)	−0.0019 (10)	0.0073 (9)	−0.0010 (9)
C11	0.0288 (14)	0.0375 (15)	0.0188 (12)	−0.0090 (12)	0.0109 (10)	−0.0013 (10)
C9	0.0203 (12)	0.0191 (12)	0.0244 (12)	−0.0036 (10)	−0.0002 (9)	0.0038 (9)
C4	0.0231 (13)	0.0139 (11)	0.0333 (13)	0.0004 (9)	0.0131 (10)	−0.0039 (9)
C3	0.0228 (12)	0.0207 (12)	0.0222 (12)	−0.0034 (10)	0.0082 (10)	−0.0063 (9)
C25	0.0235 (12)	0.0193 (12)	0.0176 (11)	0.0017 (10)	0.0048 (9)	−0.0024 (9)
C5	0.0154 (11)	0.0166 (12)	0.0295 (13)	0.0019 (9)	0.0028 (9)	0.0016 (9)
C22	0.0255 (13)	0.0276 (13)	0.0152 (11)	0.0055 (10)	0.0051 (9)	0.0029 (9)
C24	0.0248 (13)	0.0332 (15)	0.0214 (12)	0.0077 (11)	−0.0006 (10)	−0.0011 (10)
C15	0.0176 (12)	0.0147 (11)	0.0274 (12)	0.0014 (9)	−0.0048 (9)	−0.0019 (9)
C23	0.0234 (13)	0.0278 (14)	0.0211 (12)	−0.0003 (10)	0.0016 (10)	0.0019 (10)
C16	0.0282 (13)	0.0193 (12)	0.0196 (11)	0.0029 (10)	−0.0067 (10)	−0.0008 (9)
C32	0.0208 (12)	0.0185 (12)	0.0214 (12)	−0.0039 (9)	0.0064 (9)	−0.0053 (9)
B1	0.0218 (14)	0.0206 (13)	0.0173 (12)	0.0026 (11)	0.0043 (10)	−0.0017 (10)
C17	0.0282 (13)	0.0232 (12)	0.0154 (11)	0.0021 (10)	0.0040 (9)	0.0006 (9)

### Geometric parameters (Å, º)

**Table:**

Ir1—P1	2.3330 (6)	C33—H33A	0.9900
Ir1—C19	2.043 (2)	C33—H33B	0.9900
Ir1—C30	2.192 (2)	C33—C26	1.516 (3)
Ir1—C27	2.208 (2)	C33—C32	1.543 (3)
Ir1—C31	2.197 (2)	C26—H26	1.0000
Ir1—C26	2.216 (2)	C10—H10	0.9500
P1—C1	1.834 (2)	C10—C11	1.384 (4)
P1—C7	1.841 (2)	C10—C9	1.394 (4)
P1—C13	1.833 (2)	C8—H8	0.9500
F3—B1	1.389 (3)	C8—C9	1.386 (3)
F4—B1	1.386 (3)	C28—H28A	0.9900
F1—B1	1.394 (3)	C28—H28B	0.9900
F2—B1	1.380 (3)	C20—H20	0.9500
N1—N2	1.384 (3)	C21—H21A	0.9900
N1—C19	1.341 (3)	C21—H21B	0.9900
N1—C25	1.462 (3)	C21—C22	1.530 (3)
N3—C19	1.370 (3)	C12—H12	0.9500
N3—C20	1.365 (3)	C12—C11	1.392 (3)
N3—C21	1.474 (3)	C11—H11	0.9500
N2—C20	1.305 (3)	C9—H9	0.9500
C14—H14	0.9500	C4—H4	0.9500
C14—C13	1.400 (3)	C4—C3	1.392 (3)
C14—C15	1.390 (3)	C4—C5	1.389 (3)
C1—C6	1.398 (3)	C3—H3	0.9500
C1—C2	1.407 (3)	C25—H25A	0.9800
C7—C8	1.398 (3)	C25—H25B	0.9800
C7—C12	1.404 (3)	C25—H25C	0.9800
C30—H30	1.0000	C5—H5	0.9500
C30—C29	1.515 (3)	C22—H22A	0.9900
C30—C31	1.401 (3)	C22—H22B	0.9900
C13—C18	1.406 (3)	C22—C23	1.516 (3)
C27—H27	1.0000	C24—H24A	0.9800
C27—C26	1.395 (3)	C24—H24B	0.9800
C27—C28	1.518 (3)	C24—H24C	0.9800
C18—H18	0.9500	C24—C23	1.534 (3)
C18—C17	1.396 (3)	C15—H15	0.9500
C6—H6	0.9500	C15—C16	1.392 (4)
C6—C5	1.389 (3)	C23—H23A	0.9900
C2—H2	0.9500	C23—H23B	0.9900
C2—C3	1.388 (3)	C16—H16	0.9500
C29—H29A	0.9900	C16—C17	1.391 (3)
C29—H29B	0.9900	C32—H32A	0.9900
C29—C28	1.546 (3)	C32—H32B	0.9900
C31—H31	1.0000	C17—H17	0.9500
C31—C32	1.525 (3)		
			
C19—Ir1—P1	91.94 (6)	C33—C26—Ir1	109.39 (15)
C19—Ir1—C30	163.28 (8)	C33—C26—H26	114.1
C19—Ir1—C27	92.51 (8)	C11—C10—H10	120.2
C19—Ir1—C31	158.53 (9)	C11—C10—C9	119.6 (2)
C19—Ir1—C26	87.09 (8)	C9—C10—H10	120.2
C30—Ir1—P1	88.54 (6)	C7—C8—H8	119.5
C30—Ir1—C27	80.69 (8)	C9—C8—C7	121.1 (2)
C30—Ir1—C31	37.22 (8)	C9—C8—H8	119.5
C30—Ir1—C26	96.15 (8)	C27—C28—C29	112.87 (18)
C27—Ir1—P1	156.32 (6)	C27—C28—H28A	109.0
C27—Ir1—C26	36.76 (8)	C27—C28—H28B	109.0
C31—Ir1—P1	96.80 (6)	C29—C28—H28A	109.0
C31—Ir1—C27	87.32 (8)	C29—C28—H28B	109.0
C31—Ir1—C26	79.96 (8)	H28A—C28—H28B	107.8
C26—Ir1—P1	166.86 (6)	N3—C20—H20	124.0
C1—P1—Ir1	116.32 (7)	N2—C20—N3	111.9 (2)
C1—P1—C7	103.62 (10)	N2—C20—H20	124.0
C7—P1—Ir1	116.12 (7)	N3—C21—H21A	108.9
C13—P1—Ir1	112.09 (7)	N3—C21—H21B	108.9
C13—P1—C1	104.27 (10)	N3—C21—C22	113.45 (19)
C13—P1—C7	102.86 (10)	H21A—C21—H21B	107.7
N2—N1—C25	119.05 (18)	C22—C21—H21A	108.9
C19—N1—N2	113.57 (18)	C22—C21—H21B	108.9
C19—N1—C25	127.14 (19)	C7—C12—H12	119.9
C19—N3—C21	124.44 (19)	C11—C12—C7	120.2 (2)
C20—N3—C19	108.08 (18)	C11—C12—H12	119.9
C20—N3—C21	126.70 (19)	C10—C11—C12	120.7 (2)
C20—N2—N1	102.98 (18)	C10—C11—H11	119.7
N1—C19—Ir1	130.67 (16)	C12—C11—H11	119.7
N1—C19—N3	103.41 (18)	C10—C9—H9	120.0
N3—C19—Ir1	125.92 (16)	C8—C9—C10	119.9 (2)
C13—C14—H14	119.8	C8—C9—H9	120.0
C15—C14—H14	119.8	C3—C4—H4	120.1
C15—C14—C13	120.4 (2)	C5—C4—H4	120.1
C6—C1—P1	123.34 (16)	C5—C4—C3	119.9 (2)
C6—C1—C2	119.0 (2)	C2—C3—C4	120.3 (2)
C2—C1—P1	117.67 (16)	C2—C3—H3	119.8
C8—C7—P1	117.78 (17)	C4—C3—H3	119.8
C8—C7—C12	118.5 (2)	N1—C25—H25A	109.5
C12—C7—P1	123.60 (18)	N1—C25—H25B	109.5
Ir1—C30—H30	114.4	N1—C25—H25C	109.5
C29—C30—Ir1	109.27 (14)	H25A—C25—H25B	109.5
C29—C30—H30	114.4	H25A—C25—H25C	109.5
C31—C30—Ir1	71.60 (12)	H25B—C25—H25C	109.5
C31—C30—H30	114.4	C6—C5—C4	120.2 (2)
C31—C30—C29	124.6 (2)	C6—C5—H5	119.9
C14—C13—P1	120.85 (16)	C4—C5—H5	119.9
C14—C13—C18	119.33 (19)	C21—C22—H22A	108.6
C18—C13—P1	119.76 (16)	C21—C22—H22B	108.6
Ir1—C27—H27	113.8	H22A—C22—H22B	107.6
C26—C27—Ir1	71.92 (13)	C23—C22—C21	114.7 (2)
C26—C27—H27	113.8	C23—C22—H22A	108.6
C26—C27—C28	123.9 (2)	C23—C22—H22B	108.6
C28—C27—Ir1	112.53 (14)	H24A—C24—H24B	109.5
C28—C27—H27	113.8	H24A—C24—H24C	109.5
C13—C18—H18	120.0	H24B—C24—H24C	109.5
C17—C18—C13	120.1 (2)	C23—C24—H24A	109.5
C17—C18—H18	120.0	C23—C24—H24B	109.5
C1—C6—H6	119.7	C23—C24—H24C	109.5
C5—C6—C1	120.5 (2)	C14—C15—H15	120.0
C5—C6—H6	119.7	C14—C15—C16	119.9 (2)
C1—C2—H2	120.0	C16—C15—H15	120.0
C3—C2—C1	120.1 (2)	C22—C23—C24	111.7 (2)
C3—C2—H2	120.0	C22—C23—H23A	109.3
C30—C29—H29A	108.9	C22—C23—H23B	109.3
C30—C29—H29B	108.9	C24—C23—H23A	109.3
C30—C29—C28	113.38 (18)	C24—C23—H23B	109.3
H29A—C29—H29B	107.7	H23A—C23—H23B	108.0
C28—C29—H29A	108.9	C15—C16—H16	119.8
C28—C29—H29B	108.9	C17—C16—C15	120.4 (2)
Ir1—C31—H31	113.6	C17—C16—H16	119.8
C30—C31—Ir1	71.18 (12)	C31—C32—C33	112.38 (19)
C30—C31—H31	113.6	C31—C32—H32A	109.1
C30—C31—C32	124.3 (2)	C31—C32—H32B	109.1
C32—C31—Ir1	113.60 (15)	C33—C32—H32A	109.1
C32—C31—H31	113.6	C33—C32—H32B	109.1
H33A—C33—H33B	107.8	H32A—C32—H32B	107.9
C26—C33—H33A	109.0	F3—B1—F1	109.5 (2)
C26—C33—H33B	109.0	F4—B1—F3	109.9 (2)
C26—C33—C32	113.01 (18)	F4—B1—F1	107.5 (2)
C32—C33—H33A	109.0	F2—B1—F3	109.5 (2)
C32—C33—H33B	109.0	F2—B1—F4	109.5 (2)
Ir1—C26—H26	114.1	F2—B1—F1	110.8 (2)
C27—C26—Ir1	71.32 (12)	C18—C17—H17	120.1
C27—C26—C33	125.7 (2)	C16—C17—C18	119.8 (2)
C27—C26—H26	114.1	C16—C17—H17	120.1
			
Ir1—P1—C1—C6	−120.92 (17)	C30—C31—C32—C33	95.6 (3)
Ir1—P1—C1—C2	59.92 (19)	C13—P1—C1—C6	3.0 (2)
Ir1—P1—C7—C8	64.05 (18)	C13—P1—C1—C2	−176.11 (17)
Ir1—P1—C7—C12	−111.60 (18)	C13—P1—C7—C8	−58.73 (19)
Ir1—P1—C13—C14	−169.45 (15)	C13—P1—C7—C12	125.61 (19)
Ir1—P1—C13—C18	13.1 (2)	C13—C14—C15—C16	−1.6 (3)
Ir1—C30—C29—C28	37.1 (2)	C13—C18—C17—C16	−0.4 (4)
Ir1—C30—C31—C32	−106.2 (2)	C6—C1—C2—C3	−2.8 (3)
Ir1—C27—C26—C33	100.9 (2)	C2—C1—C6—C5	1.5 (3)
Ir1—C27—C28—C29	11.8 (2)	C29—C30—C31—Ir1	101.1 (2)
Ir1—C31—C32—C33	13.0 (2)	C29—C30—C31—C32	−5.1 (3)
P1—C1—C6—C5	−177.65 (17)	C31—C30—C29—C28	−43.4 (3)
P1—C1—C2—C3	176.41 (18)	C26—C27—C28—C29	94.6 (3)
P1—C7—C8—C9	−173.12 (17)	C26—C33—C32—C31	−33.8 (3)
P1—C7—C12—C11	173.78 (18)	C8—C7—C12—C11	−1.8 (3)
P1—C13—C18—C17	176.94 (17)	C28—C27—C26—Ir1	−105.4 (2)
N1—N2—C20—N3	−0.5 (2)	C28—C27—C26—C33	−4.6 (3)
N3—C21—C22—C23	−66.4 (3)	C20—N3—C19—Ir1	−179.37 (16)
N2—N1—C19—Ir1	178.98 (15)	C20—N3—C19—N1	1.0 (2)
N2—N1—C19—N3	−1.4 (2)	C20—N3—C21—C22	−48.3 (3)
C19—N1—N2—C20	1.2 (2)	C21—N3—C19—Ir1	−8.9 (3)
C19—N3—C20—N2	−0.3 (3)	C21—N3—C19—N1	171.45 (19)
C19—N3—C21—C22	143.0 (2)	C21—N3—C20—N2	−170.5 (2)
C14—C13—C18—C17	−0.6 (3)	C21—C22—C23—C24	−178.4 (2)
C14—C15—C16—C17	0.6 (4)	C12—C7—C8—C9	2.8 (3)
C1—P1—C7—C8	−167.13 (17)	C11—C10—C9—C8	−0.2 (4)
C1—P1—C7—C12	17.2 (2)	C9—C10—C11—C12	1.1 (4)
C1—P1—C13—C14	63.90 (19)	C3—C4—C5—C6	−1.9 (4)
C1—P1—C13—C18	−113.56 (18)	C25—N1—N2—C20	176.05 (19)
C1—C6—C5—C4	0.8 (3)	C25—N1—C19—Ir1	4.7 (3)
C1—C2—C3—C4	1.8 (3)	C25—N1—C19—N3	−175.69 (19)
C7—P1—C1—C6	110.38 (19)	C5—C4—C3—C2	0.6 (4)
C7—P1—C1—C2	−68.78 (19)	C15—C14—C13—P1	−175.91 (17)
C7—P1—C13—C14	−44.0 (2)	C15—C14—C13—C18	1.6 (3)
C7—P1—C13—C18	138.53 (18)	C15—C16—C17—C18	0.4 (4)
C7—C8—C9—C10	−1.8 (3)	C32—C33—C26—Ir1	37.6 (2)
C7—C12—C11—C10	−0.1 (4)	C32—C33—C26—C27	−42.9 (3)
C30—C29—C28—C27	−32.8 (3)		

### Hydrogen-bond geometry (Å, º)

**Table:**

*D*—H···*A*	*D*—H	H···*A*	*D*···*A*	*D*—H···*A*
C2—H2···F2	0.95	2.36	3.285 (3)	165
C20—H20···F1^i^	0.95	2.47	3.329 (3)	150
C25—H25*B*···F4^ii^	0.98	2.37	3.245 (3)	149

Symmetry codes: (i) −*x*+1, *y*+1/2, −*z*+1/2; (ii) *x*, −*y*+1/2, *z*+1/2.
